# The clinicopathological and prognostic value of CXCR4 expression in patients with lung cancer: a meta-analysis

**DOI:** 10.1186/s12885-022-09756-1

**Published:** 2022-06-21

**Authors:** Liping Qiu, Yuanyuan Xu, Hui Xu, Biyun Yu

**Affiliations:** Department of Pulmonary and Critical Care Medicine, Ningbo Medical Center Lihuili Hospital, 1111 Jiangnan Road, Zhejiang, 315000 China

**Keywords:** CXCR4, Lung cancer, Clinicopathological features, Prognosis, Meta-analysis

## Abstract

**Background:**

The C-X-C chemokine receptor 4 (CXCR4) has been suggested to play an important role in several types of cancers and is related to biological behaviors connected with tumor progression. However, the clinical significance and application of CXCR4 in lung cancer remain disputable. Thus, we conducted a meta-analysis to investigate the impact of CXCR4 expression on survival and clinicopathological features in lung cancer.

**Methods:**

Comprehensive literature searches were conducted in PubMed, Embase and Web of Science for relevant studies. We pooled hazard ratios (HRs)/odds ratios (ORs) with 95% confidence intervals (CIs) by STATA 12.0 to evaluate the potential value of CXCR4 expression.

**Results:**

Twenty-seven relevant articles involving 2932 patients with lung cancer were included in our meta-analysis. The results revealed that CXCR4 expression was apparently associated with poor overall survival (OS) (HR 1.61, 95% CI 1.42–1.82) and disease-free survival (HR 3.39, 95% CI 2.38–4.83). Furthermore, a significant correlation with poor OS was obvious in non-small cell lung cancer patients (HR 1.59, 95% CI 1.40–1.81) and in patients showing CXCR4 expression in the cytoplasm (HR 2.10, 95% CI 1.55–2.84) and the membrane (HR 1.74, 95% CI 1.24–2.45). CXCR4 expression was significantly associated with men (OR 1.32, 95% CI 1.08–1.61), advanced tumor stages (T3-T4) (OR 2.34, 95% CI 1.28–4.28), advanced nodal stages (N > 0) (OR 2.34, 95% CI 1.90–2.90), distant metastasis (OR 3.65, 95% CI 1.53–8.69), advanced TNM stages (TNM stages III, IV) (OR 3.10, 95% CI 1.95–4.93) and epidermal growth factor receptor (EGFR) expression (OR 2.44, 95% CI 1.44–4.12) but was not associated with age, smoking history, histopathology, differentiation, lymphatic vessel invasion or local recurrence.

**Conclusion:**

High expression of CXCR4 is related to tumor progression and might be an adverse prognostic factor for lung cancer.

## Background

Lung cancer is one of the most common tumors worldwide and a leading cause of cancer-related mortality [1]. A vast amount of progress has been made in diagnostic technology and therapeutic regimens for lung cancer. Nevertheless, the prognosis of lung cancer patients remains unsatisfactory, and only 20.5% of these patients survive for more than 5 years after diagnosis [2]. A major reason is that patients with lung cancer frequently display a high propensity for metastasis. It has been reported that more than 55% of non-small cell lung cancer (NSCLC) patients and 60% of small cell lung cancer (SCLC) patients are diagnosed after the cancer has already metastasized [2, 3]. Clinically, lung cancer can metastasize to specific target organs, such as the brain, bone, liver and adrenal glands, which is responsible for the poor prognosis [4]. Thus, the investigations on the mechanism of metastasis, as well as the identification of novel drug targets, are needed to identify patients with a high probability of metastasis and provide them with better treatments.

Emerging evidence has revealed that C-X-C chemokine receptor 4 (CXCR4), a 352 amino acid rhodopsin-like G-protein-linked receptor, is overexpressed in many different types of human cancers, including osteosarcomas [5], glioma [6], prostate cancer [7], breast cancer [8] and colorectal cancer [9]. Reportedly, high CXCR4 expression was integral to cancer cell migration and invasion [10, 11]. Although the previous studies have shown the potential prognostic value of CXCR4 in lung cancer, its actual role is still debated [12–15]. Based on this background, we performed this meta-analysis to assess the clinicopathological and prognostic significance of CXCR4 expression in patients with lung cancer.

## Methods

### Publication search

We performed a comprehensive electronic search in the PubMed, Embase and Web of Science updated to April 30th, 2020. The search terms were as follows: “lung OR pulmonary” AND “cancer OR tumor OR carcinoma OR neoplasm” AND “CXCR4 OR chemokine receptor 4”. Moreover, we manually searched the reference lists of the selected papers to identify potentially applicable studies. All clinical studies selected were written in English.

### Study inclusion and exclusion criteria

The studies obtained for our meta-analysis had to fulfill the following criteria: (1) the study had a cohort or case–control design; (2) the patients were explicitly diagnosed with lung cancer by histopathologic examinations; (3) CXCR4 expression was examined in the primary site by immunohistochemistry (IHC); and (4) publications provided sufficient information to calculate odds ratios (ORs) and 95% confidence intervals (CIs) for clinicopathological parameters or hazard ratios (HRs) and 95% CIs for overall survival (OS) and/or disease-free survival (DFS).

The exclusion criteria included the following: (1) publications that were cases, reviews, conference abstracts, patent applications, letters or non-English language papers; (2) publications only involving cell lines or animals; (3) patients who had received preoperative chemotherapy or radiotherapy; and (4) publications with duplicated data or poor quality.

### Data extraction

Two investigators extracted information from the eligible studies independently to enhance the credibility. Any disagreements were resolved by discussion and consensus. The following information was recorded: first author name, year of publication, country of origin, number of cases, clinicopathological parameters, detection method, counting method and cutoff, subcellular localization and HRs and their 95% CIs for OS or DFS. For the available articles that did not provide HRs and their 95% CIs directly, we extracted them from the Kaplan–Meier survival curves provided in the studies and calculated them with Engauge Digitizer version 4.1(http://digitizer.sourceforge.net/). Meanwhile, sufficient data were available to estimate ORs and their 95% CIs.

### Quality assessment

Two reviewers assessed the quality of the enrolled studies based on the Newcastle–Ottawa Scale (NOS) [16]. The NOS scores ranged from 0 to 9. The articles were regarded as high quality when the NOS score was greater than or equal to 6.

### Statistical analysis

All statistical analyses were performed using STATA version 12.0 (Stata Corporation, College Station, Texas, USA). We assessed the heterogeneity among the studies with the chi-squared test and *I*^2^ statistic. When *I*^2^ ≥ 50%, we chose a random-effect model for the pooled estimate; otherwise, a fixed-effect model was employed. Sensitivity analyses were performed to estimate whether any individual study influenced the results. Begg’s test was used to examine publication bias. *P* < 0.05 was considered statistically significant.

## Results

### Characteristics of the included studies

**Fig. 1:**
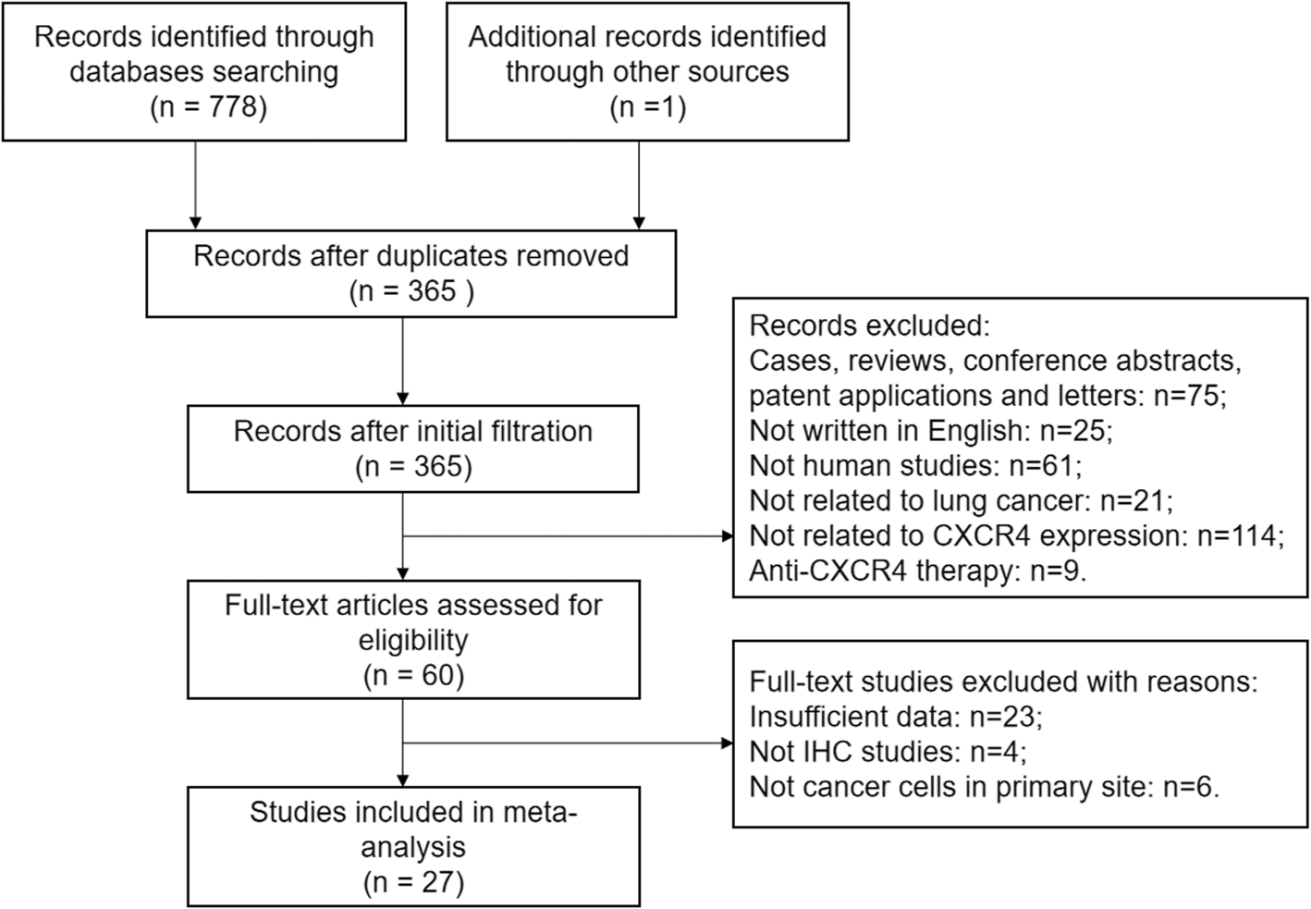
Flow chart of the literature search strategy and assessment of studies identified for meta-analysis

The details of the literature selection process are shown in Fig. 1. A total of 779 articles were initially identified by the search strategy. Then we excluded 414 duplicate articles and 306 irrelevant articles by browsing the titles and abstracts. After reviewing the full texts, 33 articles were excluded for lacking sufficient data on outcomes or clinicopathological parameters, evaluating CXCR4 expression through polymerase chain reaction and detecting CXCR4 expression only in metastatic tissues. Ultimately, 27 articles including 2932 lung cancer patients were enrolled in our meta-analysis. CXCR4 protein in lung cancer tissues was detected by IHC. The cutoff value of positive CXCR4 expression was varied among included studies. Most studies adopted a scoring system combining intensity and percentage of CXCR4 expression, while others used only intensity or percentage of CXCR4 expression. Among these studies, the R&D antibody and Abcam antibody were commonly used antibodies to against CXCR4. The basic characteristics are presented in Table 1.

**Table 1:** Characteristics of the included studies

First author (year)	Country	Time span	Case number (low/high)	Histological type	TNM	Antibody source	Counting method	Cutoff (positive)	Subcellular localization	Outcome	NOS score
Takanami (2003)	Japan	1992–1996	71(29/42)	NSCLC	I-III	R&D	P	10%	membrane and/or cytoplasm	NA	6
Spano (2004)	France	1987–1999	61(44/17)	NSCLC	I	Abcam	S + P	scores = 6/9	nucleus	OS^a^	8
Su (2005)	China	NA	34 (17/17)	NSCLC	I-III	R&D	S	≥ normal	membrane and/or cytoplasm	NA	6
Na (2008)	Germany	NA	46 (24/22)	NSCLC	I-IV	Abcam	S	scores = 3	cytoplasm/nucleus	NA	6
Song (2008)	Korea	1995–1999	323 (275/48)	NSCLC	I-IV	Abcam	P	> 50%	cytoplasm and nucleus	OS/DFS^a^	7
Suzuki (2008)	Japan	1995–2000	90(68/22)	NSCLC	I-IV	Santa Cruz	P	≥ 10%	membrane and/or cytoplasm	OS^a^	6
Wagner (2009)	America	NA	154(92/62)	NSCLC	I-IV	R&D	S	scores ≥ 2	membrane/nucleus	DFS	7
Chen (2011)	China	1998.1–2008.6	64(13/51)	NSCLC	I-IV	Abcam	S + P	scores ≥ 3	membrane and/or cytoplasm	NA	8
Otsuka (2011)	Canada	2003–2006	170(141/29)	NSCLC	IV	Abcam	AQUA	scores ≥ 3371	cytomembrane	OS^a^	8
Wang (2011)	China	2002–2004	208(91/117)	NSCLC	I-III	R&D	S + P	scores ≥ 2	cytoplasm	OS	8
Zhou (2012)	China	2002.6–2006.12	105(33/62)	NSCLC	III	Boao Sen	S + P	scores ≥ 4	cytoplasm	NA	6
Al Zobair (2013)	China	NA	125(63/62)	NSCLC	I-IV	Abcam	S + P	scores ≥ 2	cytoplasm	OS	8
Li (2014)	China	1999–2009	50(15/35)	SCLC	I-IV	R&D	S + P	scores > 2	membrane and/or cytoplasm	OS^a^	9
Wang (2014)	China	1998.1–2008.1	105(42/63)	NSCLC	I-IV	Abcam	S + P	scores ≥ 4	NA	NA	7
Kaemmerer (2015)	Germany	1998–2011	90(23/47)	BP-NEN	NA	UMB-2	S + P	scores ≥ 5	membrane	OS^a^	8
Li (2015)	China	2003.6–2009.10	65(31/34)	SCLC	I-III	Abcam	S + P	scores ≥ 6	cytoplasm	OS^a^	6

*NSCLC* Non-small cell lung cancer, *BP-NENS* Bronchopulmonary neuroendocrine neoplasms, *AC* Adenocarcinoma, *ASC* Adenosquamous carcinoma, *S* Staining intensity, *P* Percentage of positively-stained cells, *HR* Hazard ratio

^a^extracted from the Kaplan–Meier survival curves, *OS* Overall survival, *DFS* Disease-free survival, *DSS* Disease specific survival, *NOS* Newcastle–Ottawa Scale, *NA* Not available

### CXCR4 expression and outcome

**Fig. 2:**
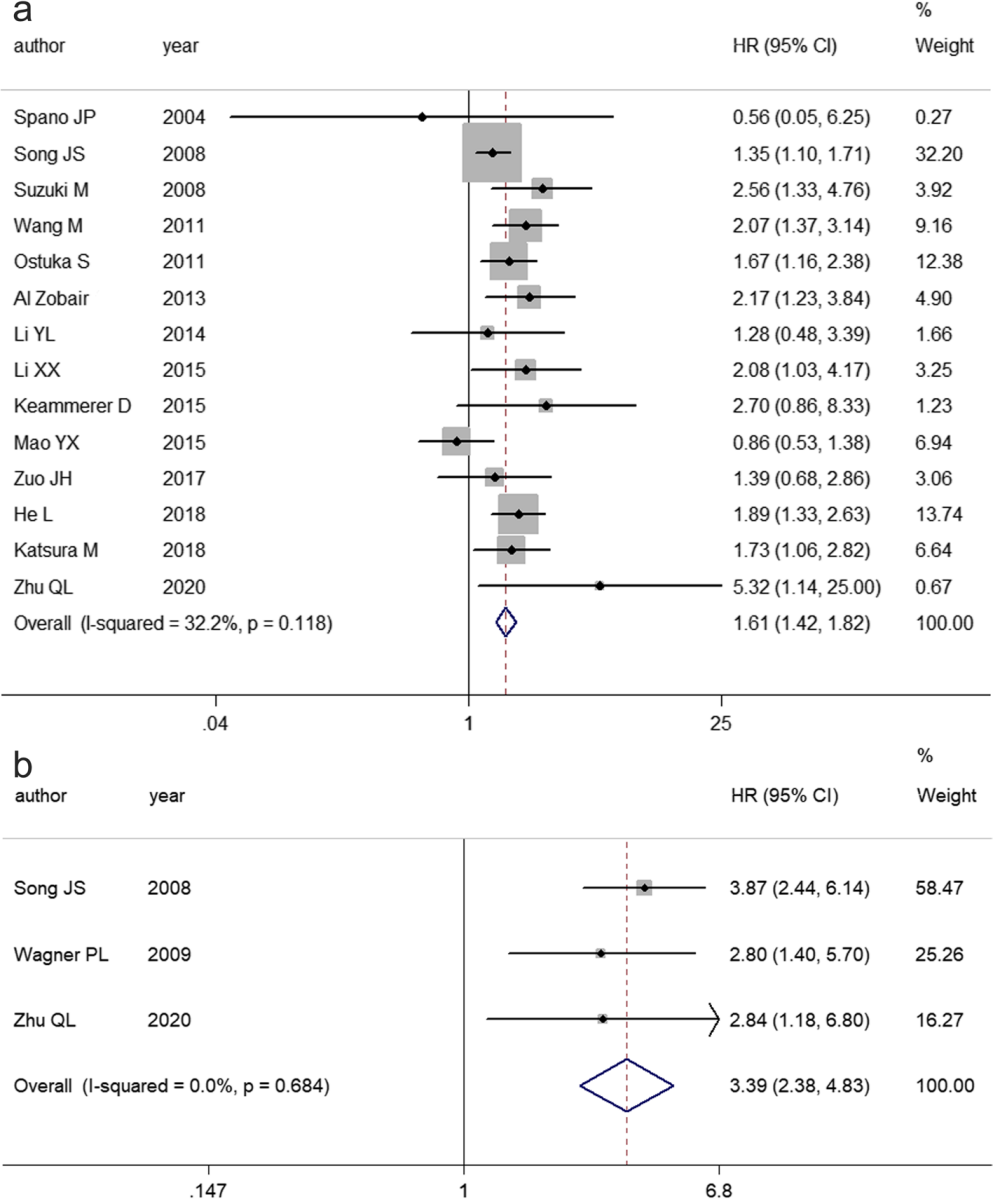
Forest plots for the association between CXCR4 expression and **a** OS, **b** DFS

Fourteen out of 27 studies including 1899 patients with lung cancer evaluated the association between CXCR4 expression and overall survival (OS) [12–15, 17–26]. The pooled HR showed that high CXCR4 expression was linked to decreased OS in lung cancer (HR 1.61, 95% CI 1.42–1.82, *P* < 0.001, *I*^2^ = 32.2%) (Fig. 2a).

As shown in Table 2, the stratified analysis by histology demonstrated that high expression of CXCR4 predicted unfavorable OS in both NSCLC (HR 1.59, 95% CI 1.40–1.81, *P* < 0.001, *I*^2^ = 43.2%) and SCLC patients (HR 1.77, 95% CI 1.00–3.12, *P* = 0.050, *I*^2^ = 0). However, the latter did not achieve statistical significance. In addition, increased CXCR4 expression in the membrane (HR 1.74, 95% CI 1.24–2.45, *P* < 0.001, *I*^2^ = 0) and cytoplasm (HR 2.10, 95% CI 1.55–2.84, *P* < 0.001, *I*^2^ = 0) was significantly associated with poor OS, while its expression in the nucleus was associated with favorable prognosis (HR 0.56, 95% CI 0.05–6.25). The HR from 7 studies including early resected lung cancer (stage I-III) patients and 1 study including metastatic lung cancer (stage IV) patients showed that increased CXCR4 expression predicted poor OS (HR 1.62, 95% CI 1.32–1.98, *P* < 0.001, *I*^2^ = 48.5%; HR 1.67, 95% CI 1.16–2.38). The prognostic effects were similar between the subgroups by geographical area, NOS scores and statistical analysis (Table 2).

**Table 2:** Subgroup analysis of the association between CXCR4 expression and OS according to different parameters

parameters	No. of studies	Cases	HR (95% CI) of OS	*P*	*I*^2^(%)	Effect model
**Histological type**						
NSCLC	11	1540	1.59(1.40–1.81)	< 0.001	43.2	Fixed
SCLC	2	115	1.77(1.00–3.12)	0.050	0	Fixed
**Geographical area**						
non-Asian	3	321	1.70(1.22–2.39)	0.002	0	Fixed
Asian	11	1424	1.57(1.26–1.96)	< 0.001	43.1	Fixed
**NOS score**						
≥ 7	7	495	1.99(1.42–2.78)	< 0.001	0	Fixed
< 7	7	1250	1.59(1.30–1.96)	< 0.001	50.5	Random
**Subcellular localization**						
membrane	2	260	1.74(1.24–2.45)	< 0.001	0	Fixed
cytoplasm	3	438	2.10(1.55–2.84)	< 0.001	0	Fixed
nucleus	1	61	0.56(0.05–6.25)	-	-	-
**TNM stage**						
Stage I-III	7	680	1.62(1.32–1.98)	< 0.001	48.5	Fixed
Stage IV	1	170	1.67(1.16–2.38)	-	-	-
**Statistical analysis**						
Univariate analysis	9	883	1.81(1.50–2.17)	< 0.001	0	Fixed
Multivariate analysis	5	862	1.59(1.09–2.31)	0.016	67.8	Random

*HR* Hazard ratio, *OS* Overall survival, *NSCLC* Non-small cell lung cancer, *NOS* Newcastle–Ottawa Scale

In addition, only 3 studies including 555 patients with resected lung cancer were enrolled to pool the HR for DFS [13, 26, 27]. All patients were treated with initial surgical resection. Using a fixed-effects model, the results showed that increased CXCR4 expression was associated with reduced DFS in lung cancer (HR 3.39, 95% CI 2.38–4.83, *P* < 0.001, *I*^2^ = 0%) (Fig. 2b).

### CXCR4 expression and clinicopathological features

**Fig. 3:**
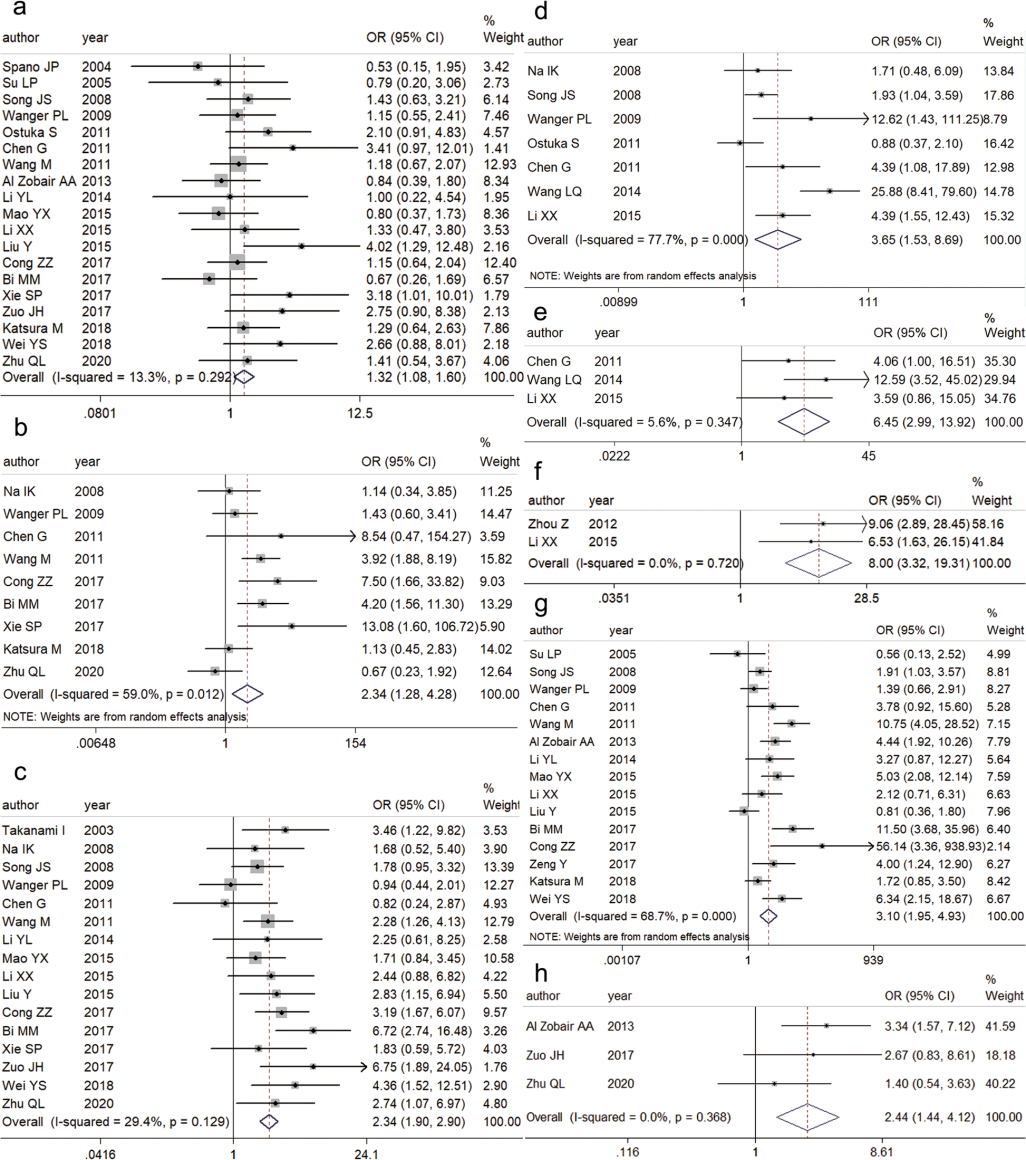
Forest plots for the association between CXCR4 expression and **a** sex (female vs. male), **b** tumor stage (T1, 2 vs. T3, 4), **c** nodal stage (N0 vs. N > 0), **d** distant metastasis (M0 vs. M1), **e** brain metastasis (no vs. yes), **f** bone metastasis (no vs. yes), **g** TNM stage (I, II vs. III, IV), **h** EGFR expression (low vs. high)

To identify the pathological diagnostic value of CXCR4 expression, we investigated the association between CXCR4 expression and clinicopathological features (Table 3) [12, 13, 15, 18–21, 27–35]. The pooled OR from 19 studies including 2208 patients revealed a significant six-dependent difference in CXCR4 expression through a fixed-effects model (OR 1.32, 95% CI 1.08–1.61, *P* = 0.006) (Fig. 3a). Nine out of 23 studies including 1049 patients examined the association between CXCR4 expression and tumor category. The pooled OR, calculated by a random-effects model, for the T1-T2 group versus the T3-T4 group was 2.34 (95% CI 1.28–4.28, *P* = 0.006) (Fig. 3b). Sixteen studies including 1795 patients showed a statistically significant association between CXCR4 expression and lymph node metastasis (OR 2.35, 95% CI 1.90–2.90, *P* < 0.001) (Fig. 3c). The pooled OR of 7 studies with substantial heterogeneity (*I*^2^ = 70.5%) indicated that CXCR4 expression was increased in lung cancer with distant metastasis compared to lung cancer without distant metastasis (OR 3.65, 95% CI 1.53–8.68, *P* = 0.003) (Fig. 3d). Using a fixed-effects model, the pooled OR of 3 studies revealed a significant association between CXCR4 expression and brain metastasis (OR 6.45, 95% CI 2.99–13.92, *P* < 0.001) (Fig. 3e). The association of CXCR4 expression with bone metastasis was also investigated in two studies through a fixed-effects model (OR 8.00, 95% CI 3.32–19.31, *P* < 0.001) (Fig. 3f). Meanwhile, a random-effects model revealed that elevated CXCR4 expression was more frequently observed in advanced stages (III, IV) than those in early stages (I, II) (OR 3.10, 95% CI 1.95–4.93, *P* < 0.001) (Fig. 3g). Fixed-effects models showed that the differences in CXCR4 expression between stage I and stage II (OR 1.50, 95% CI 1.11–2.03, *P* = 0.008), stage II and stage III (OR 2.76, 95% CI 2.01–3.78, *P* < 0.001), and III and stage IV (OR 4.44, 95% CI 2.098–9.398, *P* < 0.001) were statistically significant. Furthermore, we investigated the association of high CXCR4 expression with increased epidermal growth factor receptor (EGFR) expression. Three studies showed that increased CXCR4 expression levels were likely to be associated with EGFR expression (OR 2.44, 95% CI 1.44–4.12, *P* = 0.001) in a fixed-effects model (Fig. 3h).

**Table 3:** Pooled OR (95% CI) of association of CXCR4 expression with clinicopathological indicators

clinicopathological features	No. of studies	Cases	Pooled OR (95% CI)	*P*	*I*^*2*^(%)	Effect model
Age (< 60 vs. ≥ 60)	4	670	0.76(0.53–1.09)	0.136	0	Fixed
Sex (female vs. male)	19	2208	1.32(1.08–1.61)	0.006	13.3	Fixed
Smoking history (never vs. former/current)	4	589	1.38 (0.95–2.00)	0.095	3.0	Fixed
Tumor stage (T1, 2 vs. T3, 4)	9	1049	2.34(1.28–4.28)	0.006	59.0	Random
Nodal stage (N0 vs. N > 0)	16	1795	2.34(1.90–2.90)	< 0.001	29.4	Fixed
Distant metastasis (M0 vs. M1)	7	922	3.65 (1.53–8.68)	0.003	77.7	Random
Brain Metastasis (no vs. yes)	3	234	6.45 (2.99–13.92)	< 0.001	5.6	Fixed
Bone Metastasis (no vs. yes)	2	170	8.00 (3.32–19.31)	< 0.001	0	Fixed
TNM stage (I, II vs. III, IV)	15	1833	3.10(1.95–4.93)	< 0.001	68.7	Random
TNM stage (I vs. II)	11	963	1.50(1.11–2.03)	0.008	31.3	Fixed
TNM stage (II vs. III)	12	900	2.76(2.01–3.78)	< 0.001	33.0	Fixed
TNM stage (III vs. IV)	5	588	4.44(2.10–9.40)	< 0.001	0	Fixed
Histological type (non-SCC vs. SCC)	15	1829	1.12(0.82–1.63)	0.405	54.8	Random
Differentiation (well/moderate vs. poor)	12	1310	0.90 (0.57,1.42)	0.647	58.4	Random
EGFR expression (low vs. high)	3	264	2.44 (1.44,4.12)	0.001	0	Fixed
lymphatic vessel invasion (no vs. yes)	3	401	1.42(0.39–5.21)	0.599	88.1	Random
Local recurrence (no vs. yes)	2	383	1.18 (0.49–2.85)	0.720	0	Fixed

*OR* Odds ratio, *EGFR* Epidermal growth factor receptor expression, *SCC* Squamous cell carcinoma

However, no statistically significant association was observed between CXCR4 expression and age (< 60 vs. ≥ 60 years) (OR 0.76, 95% CI 0.53–1.09, *P* = 0.136), smoking history (never vs. former/current) (OR 1.38, 95% CI 0.95–2.00, *P* = 0.095), histological type (non-SCC vs. SCC) (OR 1.12, 95% CI 0.82–1.63, *P* = 0.405), differentiation status (well/moderate vs. poor) (OR 0.90, 95% CI 0.57–1.42, *P* = 0.647), lymphatic vessel invasion (no vs. yes) (OR 1.42, 95% CI 0.39–5.21, *P* = 0.599) or local recurrence (no vs. yes) (OR 1.18, 95% CI 0.49–2.85, *P* = 0.720) (Table 3).

The increased CXCR4 expression was related to the clinicopathological features of lung cancer, but high heterogeneity was observed for the associations with tumor stage (*I*^*2*^ = 59.0%), distant metastasis (*I*^*2*^ = 77.7%), and TNM stage (*I*^*2*^ = 68.7%). To elucidate the sources of heterogeneity, we performed subgroup analysis based on geographical area and subcellular localization (Table 4). As seen in Table 4, the results showed that elevated CXCR4 expression was associated with advanced tumor stages (OR 2.95, 95% CI 1.37–6.34, *P* = 0.006, *I*^*2*^ = 64.1%) and distant metastasis (OR 5.33, 95% CI 1.68–16.94, *P* = 0.005, *I*^*2*^ = 82.1%) in the Asian group. However, the amount of heterogeneity was still relatively large. There was no association of CXCR4 expression with tumor stage (OR 1.32, 95% CI 0.65–2.68, *P* = 0.438, *I*^*2*^ = 0) or distant metastasis (OR 1.93, 95% CI 0.56–6.72, *P* = 0.301, *I*^*2*^ = 61.4) in the non-Asian group. In the staining pattern subgroup analysis, CXCR4 localization impacted the association between increased CXCR4 expression and advanced tumor stages with decreased heterogeneity (membrane and/or cytoplasm: OR 7.76, 95% CI 2.03–29.69, *P* = 0.003, *I*^*2*^ = 0; cytoplasm: OR 4.02, 95% CI 2.23–7.26, *P* < 0.001, *I*^*2*^ = 0; membrane and nucleus: OR 1.28, 95% CI 0.68–2.40, *P* = 0.446, *I*^*2*^ = 0) as well as between increased CXCR4 expression and advanced TNM stages (membrane and/or cytoplasm: OR 2.75, 95% CI 1.15–6.56, *P* = 0.023, *I*^*2*^ = 70.7%; cytoplasm: OR 6.18, 95% CI 3.98–9.59, *P* < 0.001, *I*^*2*^ = 40.1%; membrane and nucleus: OR 1.56, 95% CI 0.94–2.59, *P* = 0.088, *I*^*2*^ = 0). The results indicated that elevated CXCR4 expression in the group of membrane and/or cytoplasm group and the cytoplasm group was significantly associated with advanced tumor stages and TNM stages. In addition, significantly increased CXCR4 expression in the cytoplasm and nucleus was associated with distant metastasis (OR 1.89 95% CI 1.08–3.30, *P* = 0.867, *I*^*2*^ = 0), but this association as not seen for increased CXCR4 expression in the membrane and/or cytoplasm (OR 1.78 95% CI 0.37–8.56, *P* = 0.056, *I*^*2*^ = 72.7%). Moreover, different CXCR4 localizations and geographical areas might be resources causing the high heterogeneity of tumor stage. The high heterogeneity of distant metastasis and TNM stage was likely caused by different CXCR4 localizations.

**Table 4:** Subgroup analysis of the association between CXCR4 expression and clinicopathological indicators

parameters	No. of studies	Cases	Pooled OR (95% CI)	*P*	*I*^2^(%)	Effect model
**Tumor stage**						
***Geographical area***						
non-Asian	2	200	1.32(0.65–2.68)	0.438	0	Fixed
Asian	7	849	2.95(1.37–6.34)	0.006	64.1	Random
***Subcellular localization***						
membrane and/or cytoplasm	2	249	7.76(2.03–29.69)	0.003	0	Fixed
cytoplasm	2	318	4.02(2.23–7.26)	< 0.001	0	Fixed
membrane and nucleus	2	294	1.28(0.68–2.40)	0.446	0	Fixed
**Distant metastasis**						
***Geographical area***						
non-Asian	3	370	1.93(0.56–6.72)	0.301	61.4	Random
Asian	4	552	5.33(1.68–16.94)	0.005	81.2	Random
***Subcellular localization***						
membrane and/or cytoplasm	2	234	1.78(0.37–8.56)	0.056	72.7	Random
cytoplasm and nucleus	2	364	1.89(1.08–3.30)	0.867	0	Fixed
**TNM stage**						
***Subcellular localization***						
membrane and/or cytoplasm	7	641	2.75(1.15–6.56)	0.023	70.7	Random
cytoplasm	5	576	6.18(3.98–9.59)	0	40.1	Fixed
membrane and nucleus	2	294	1.56(0.94–2.59)	0.088	0	Fixed

*OR* Odds ratio

### Sensitivity analysis

Sensitivity analysis in which individual studies were omitted sequentially was performed using a random-effects model. Finally, after omitting the study from Otsuka [18], the heterogeneity of histology was no longer observed (*I*^2^ = 15.1%). We found no significant heterogeneity in other sensitivity analyses.

### Publication bias

Publication biases were assessed by Begg’s tests. The results did not present apparent publication bias among the studies regarding OS (*P* = 0.743), DFS (*P* = 1.000), age (*P* = 0.308), sex (*P* = 0.168), smoking history (*P* = 0.308), tumor stage (*P* = 0.917), nodal stage (*P* = 0.893), distant metastasis (*P* = 0.230), brain metastasis (*P* = 0.296), bone metastasis (*P* = 1.000), TNM stage (*P* = 0.373), histology (*P* = 0.373), differentiation (*P* = 0.837), EGFR expression (*P* = 1.000), lymphatic vessel invasion (*P* = 0.296) or local recurrence (*P* = 1.000).

## Discussion

CXCR4, a type of chemokine receptors, is widely expressed in malignant tumors and its oncogenic role has been confirmed in various cancers [5, 8]. A few researchers detected CXCR4 expression in tumor cells and tumor-infiltrating lymphocytes but scarcely in normal lung tissues [13, 27, 36]. Wald et al*.* [37] found that CD4^+^ T cells expressed high levels of CXCR4 expression compared with CD8^+^ T cells and NK cells in lung adenocarcinoma tumors, which might contribute to suppression of the immune response against tumor. The CD4^+^CD25^high^FoxP3^+^ regulatory T cells, associated with tumor progression, were shown to express CXCR4 and be recruited into lung cancer [37, 38]. Franco et al*.* [39] reported that CXCR4 expression in tumor cells was associated with increased microvascular density and microvessel invasion. C-X-C motif chemokine ligand 12 (CXCL12), also known as the sole ligand of CXCR4, has been verified to be expressed in many tissues and cell types [40] Previous studies demonstrated that the positive expression rate of CXCL12 in lung cancer was 31.3–80% [14, 17, 27, 34]. In tumor microenvironment, CXCL12 could be expressed by tumor, immune and stromal cells [41]. Sterlacci et al*.* [42] proposed that CXCL12 expression in stromal cells and tumor cells was associated with the activated form of CXCR4 expression by tumor cells. Meanwhile, CXCL12 expression in tumor promoted the recruitment of CXCR4-expressing immune cells to potentiate the tumor-promoting effect [37]. In addition, CXCL12 expression, especially in tumor cell membrane, was correlated with metastasis [42]. Some studies revealed that CXCL12 protein expression levels were significantly higher in metastatic sites than in primary tumor site, which mediated the metastasis of CXCR4-expressing tumor cells in lung cancer [25, 43]. Hence, CXCL12/CXCR4 axis triggers downstream signaling pathways and plays a vital role in proliferation, angiogenesis, migration and therapeutic resistance [44, 45]. Inhibiting CXCL12/CXCR4 axis by CXCR4 antagonists can be a value treatment option in lung cancer. However, CXCR4 expression in lung cancer is controversial. Previous meta-analyses have described the association between CXCR4 expression and NSCLC [46, 47]. Considering the availability of novel studies including a greater number of patients, we performed an updated meta-analysis to evaluate the clinicopathological and prognostic value of CXCR4 expression in lung cancer.

According to previous studies, CXCR4 was overexpressed in 52.3-100% of SCLC patients and 14.9-79.7% of NSCLC patients [13, 21, 22, 30, 48]. Stumpf et al. [48] found that CXCR4 expression intensity was distinctly higher in SCLC than in squamous cell carcinoma (*P* = 0.002) and adenocarcinoma (*P* = 0.001) by using the Mann–Whitney test. Regarding the prognostic value of CXCR4 expression, the results of our meta-analysis demonstrate that elevated CXCR4 expression appears to be related to poorer OS in lung cancer. Moreover, we found that CXCR4 upregulation was a prognostic factor for unfavorable OS in both early resected and metastatic patients with lung cancer. Stratified analysis by histology showed that increased CXCR4 expression was significantly associated with poor OS in NSCLC patients. However, the prognostic effect of CXCR4 expression in SCLC patients did not reach statistical significance in our analysis. A previous study showed that SCLC patients with urokinase-type plasminogen activator receptor (uPAR) and CXCR4 coexpression had shorter OS than those with single and co-negative uPAR or CXCR4 expression (*P* = 0.033) [20]. We speculate that CXCR4 expression synergizes with other molecules to influence the prognosis of SCLC. To date, the studies investigating the expression of CXCR4 in SCLC have been relatively limited. Further studies with a larger sample of patients are needed. Our analysis combining the outcomes of 555 resected NSCLC patients from 3 individual studies indicated that high CXCR4 expression significantly predicted poor DFS.

CXCR4 has been identified in every subcellular localization. Spano et al. [12] and Wanger et al. [27] found that strong nuclear CXCR4 staining was associated with a better outcome in lung cancer, whereas cytomembrane CXCR4 staining was significantly associated with decreased DFS in Wagner’s study The prognostic role of cytoplasmic CXCR4 expression has not reached a consensus. Shi et al. [49] reported that aberrant cytoplasmic CXCR4 expression predicted a favorable outcome in triple-negative breast cancer patients. An animal experiment showed that the retention of CXCR4 in the endoplasmic reticulum of T-cell hybridoma could reduce metastasis and improve the prognosis of mice [50]. In contrast, Wang et al. [14] reported that high cytoplasmic CXCR4 staining was an adverse prognostic factor for lung cancer. Thus, different subcellular localizations of CXCR4 expression might lead to different biological behaviors and might have clinical application value. Our subgroup analysis indicated that cytoplasmic and membrane CXCR4 staining conferred a more significant association with poor OS than nuclear CXCR4 staining in lung cancer. It could be speculated that nuclear CXCR4 localization inhibited its signal transduction pathway and that heterotopic CXCR4 promoted the progression of tumors [12]. Franco et al. [39] demonstrated that high cytoplasmic and membranous CXCR4 expression in tumor cells significantly increased microvascular density and microvessel invasion in NSCLC. In addition, Saba et al. [51] discovered that cytoplasmic CXCR4 was associated with the loss of epithelial markers and the activation of intracellular signaling pathways in NSCLC, which might promote epithelial-mesenchymal transition and tumor progression.

In addition, we found that aberrant CXCR4 expression in primary cancerous tissue was strongly correlated with adverse prognostic factors at diagnosis, such as male sex and advanced TNM stages. Consistent with previous studies, our pooled results showed an increase in CXCR4 expression with clinical stage progression which suggested that CXCR4 expression was closely associated with the invasion and metastasis of tumors [14, 15, 19, 35, 52]. A study by Zeng et al. [53] verified that upregulating CXCR4 expression significantly increased the metastatic ability of lung cancer cells in experimental studies. To exclude the influence of these variables on the association between CXCR4 expression and outcome, we conducted a subgroup analysis of statistical analysis. Our results showed that high CXCR4 expression, whether in multivariate analysis or in univariate analysis, was associated with poor survival in patients with lung cancer. As multivariate analysis is an effective method for reducing bias from various confounding variables and making statistically reliable conclusions [54], we reasoned that CXCR4 overexpression might be an independent prognostic factor in lung cancer.

Additionally, the association between CXCR4 expression and advanced tumor staging was more apparent in Asian patients than in non-Asian patients. Genomic polymorphisms and different environmental exposures may explain this difference. The underlying mechanism of CXCR4 expression in tumor progression and metastasis has been investigated over the years. A study conducted by Paratore et al. [55] showed that CXCR4/CXCL12 immunoreactivities in NSCLC with brain metastases were significantly higher than those in paired NSCLC without brain metastases. Chen et al. [30] indicated a similar result and suggested that CXCL12 expression in the brain might mediate the homing of lung cancer cells with high CXCR4 expression. Liao et al. [56] confirmed that CXCL12 stimulated CXCR4 expression and then increased soluble vascular cell adhesion molecule 1 (sVCAM1) secretion in NSCLC, which could recruit and arrest osteoclast progenitors to promote osteoclastogenesis in metastatic bone tissue. A similar result was found in the SCLC population [21]. Studies have reported that tumor metastasis target tissues frequently express high levels of CXCL12 [57]. In this setting, CXCL12 could establish a chemotactic gradient between the primary and metastatic sites, facilitating the transfer of CXCR4-positive cancer cells into tissues rich in CXCL12. Hence, we speculated that CXCR4 might be a sensitive marker for predicting metastatic diseases.

Our results indicated that high CXCR4 expression was significantly associated with epidermal growth factor receptor (EGFR) overexpression. EGFR is a receptor tyrosine kinase expressed on the epithelial cells [58]. Previous studies confirmed that EGFR can enhance the expression of CXCR4 in some cancers, including breast and ovarian cancers [59, 60]. The potential mechanism found in experimental research was that EGFR activation could activate its downstream PI3K/AKT signaling pathway and subsequently stimulate CXCR4 expression [61]. Additionally, Al Zobair et al. [19] found that patients with EGFR/CXCR4 dual expression had significantly shorter OS than those with single positive expression or dual negative expression (HR 2.42, 95% CI 1.40-4.17, *P* = 0.010). On the basis of these findings, we proposed that the subpopulation with the concomitant expression of CXCR4/EGFR was indeed worthy of attention.

Previous studies have verified that CXCR4 antagonists produce therapeutic effects in many diseases varying from cancers to human immunodeficiency virus (HIV) [62, 63]. Lee et al. [64] found that blocking the CXCL12/CXCR4 axis with anti-CXCR4 antibodies could decrease breast cancer cell migration to the brain. It has been reported in an experimental study that blocking CXCR4 inhibited the proliferation of lung cancer cells and the migration to CXCL12 [65]. In recent experimental studies, CXCR4 expression was shown to mediate cisplatin resistance in a cytochrome P450 1B1 (CYP1B1)-dependent manner way and paclitaxel resistance by increasing the expression of antiapoptotic proteins [26, 66]. Another study showed that a CXCR4 antagonist significantly suppressed acquired resistance to gefitinib in a lung adenocarcinoma cell line harboring EGFR mutations [67]. A study conducted by D'Alterio et al. [68] demonstrated that CXCR4 antagonists could reshape tumor microenvironment favoring access of T effector and reducing regulatory T cells to intensify the efficacy of anti-programmed death 1 therapy. Overall, we hypothesized that CXCR4 inhibitors could improve the prognosis of patients with lung cancer by preventing the distal metastasis of tumor cells and improving the therapeutic effect of conventional therapy or immunotherapy. To date, 9 kinds of CXCR4 antagonists are currently in clinical trials or were in completed clinical trials [69]. However, the applications of CXCR4 antagonists in lung cancer are relatively limited and need further exploration.

Nevertheless, this meta-analysis should be interpreted in view of certain limitations. First, most studies employed a semiquantitative scoring system by combining the intensity and proportion of the stained tumor cells. However, there was still a difference in defining the percentage of positively stained cells among the studies. There were not enough data for us to perform subgroup analysis by the same cutoff and antibody to analyze the underlying bias. Second, we calculated the HRs and 95% CIs from Kaplan-Meier curves in the majority of studies, which might reduce the accuracy of the results. Third, most enrolled studies were performed in Asia and our subgroup analysis indicated that geographical area might be a cause of heterogeneity. Thus, more research involving other populations is needed to further confirm the value of CXCR4 expression in lung cancer.

## Conclusion

Our meta-analysis suggested that high CXCR4 expression could serve as a promising predictive marker for poor prognosis in lung cancer. In addition, increased CXCR4 expression was more common in men and was associated with advanced stages, metastasis and EGFR expression. CXCR4 antagonists combined with conventional therapy or immunotherapy may enhance the treatment efficacy and improve the prognosis of patients with lung cancer. Further large-scale studies are needed to confirm the current results.

## Data Availability

All data generated or analyzed during this study are included in this published article.

## Abbreviations

AC: Adenocarcinoma
ASC: Adenosquamous carcinoma
CI: Confidence interval
CXCL12: C- X-C motif ligand 12
CXCR4: C-X-C chemokine receptor 4
CYP1B1: Cytochrome P450 1B1
DFS: Disease free survival
EGFR: Epidermal growth factor receptor
HIF-1a: Hypoxia inducible factor-1a
HIV: Human immunodeficiency virus
HR: Hazard ratio
IHC: Immunohistochemistry
NOS: Newcastle–Ottawa Scale
NSCLC: Non-small cell lung cancer
OR: Odds ratio
OS: Overall survival
uPAR: Urokinase-type plasminogen activator receptor (uPAR)
SCC: Squamous cell carcinoma
SCLC: Small cell lung cancer
sVCAM1: Soluble vascular cell adhesion molecule 1
TNM: Tumor-node-metastasis
PTX: Paclitaxel
